# The endogenous production of hydrogen sulphide in intrauterine tissues

**DOI:** 10.1186/1477-7827-7-10

**Published:** 2009-02-06

**Authors:** Pushpa Patel, Manu Vatish, John Heptinstall, Rui Wang, Ray J Carson

**Affiliations:** 1 College of Medical & Dental Sciences, University of Birmingham, Birmingham, UK; 2 Molecular Medicine Research Group, University of Warwick, Coventry, UK; 3 Biomolecular Sciences Dept., Coventry University, Coventry, UK; 4 Department of Biology, Lakehead University, Thunder Bay, Ontario, Canada; 5 Dept of Medical & Social Care Education, School of Medicine, University of Leicester, Leicester, UK

## Abstract

**Background:**

Hydrogen sulphide is a gas signalling molecule which is produced endogenously from L-cysteine via the enzymes cystathionine beta-synthase (CBS) and cystathionine gamma-lyase (CSE). The possible role of hydrogen sulphide in reproduction has not yet been fully investigated. It has been previously demonstrated that hydrogen sulphide relaxes uterine smooth muscle in vitro. The aim of the present study was to investigate the endogenous production of hydrogen sulphide in rat and human intrauterine tissues in vitro.

**Methods:**

The production of hydrogen sulphide in rat and human intrauterine tissues was measured in vitro using a standard technique. The expression of CBS and CSE was also investigated in rat and human intrauterine tissues via Western blotting. Furthermore, the effects of nitric oxide (NO) and low oxygen conditions on the production rates of hydrogen sulphide were investigated.

**Results:**

The order of hydrogen sulphide production rates (mean +/- SD, n = 4) for rat tissues were: liver (777 +/- 163 nM/min/g) > uterus (168 +/- 100 nM/min/g) > fetal membranes (22.3 +/- 15.0 nM/min/g) > placenta (11.1 +/- 4.7 nM/min/g), compared to human placenta (200 +/- 102 nM/min/g). NO significantly increased hydrogen sulphide production in rat fetal membranes (P < 0.05). Under low oxygen conditions the production of hydrogen sulphide was significantly elevated in human placenta, rat liver, uterus and fetal membranes (P < 0.05). Western blotting (n = 4) detected the expression of CBS and CSE in all rat intrauterine tissues, and in human placenta, myometrium, amnion and chorion.

**Conclusion:**

Rat and human intrauterine tissues produce hydrogen sulphide in vitro possibly via CBS and CSE enzymes. NO increased the production of hydrogen sulphide in rat fetal membranes. The augmentation of hydrogen sulphide production in human intrauterine tissues in a low oxygen environment could have a role in pathophysiology of pregnancy.

## Background

The pharmacological, physiological and pathological roles of gasotransmitters nitric oxide (NO) and carbon monoxide (CO) have been extensively researched in the reproductive system. NO donors have been demonstrated to relax the myometrium [1] and maintain uterine quiescence [2]. CO has been demonstrated to relax smooth muscle, including human myometrium via a sGC-cGMP mechanism [3]. Hydrogen sulphide (H_2_S) is another gasotransmitter that has many parallels with NO and CO [4], however there are no reports to date on the production of H_2_S in reproductive tissues. H_2_S is endogenously produced from L-cysteine by two pyridoxal 5' phosphate-dependent enzymes cystathionine β-synthase (CBS) and cystathionine γ-lyase (CSE) [5-7]. A high expression of CBS has been found in the central nervous system [8] while CSE is highly expressed in vascular tissues in the rat [9]. An early study by Smythe [10] observed the production of H_2_S in rat liver from L-cysteine. In more recent studies various mammalian tissues have been shown to produce H_2_S, including the brain (50–160 μM), the ileum, the kidneys and vascular tissue [8,11,12].

The physiological roles of H_2_S have been well established. Abe and Kimura [8] suggested possible role of H_2_S as an endogenous neuromodulator in rat brain tissue, where it is involved in synaptic transmission. In the cardiovascular system H_2_S acts as a vasodilator both in vivo and in vitro and reduces blood pressure in vivo. The mechanism of action of H_2_S is unknown. However, unlike NO, H_2_S dilates blood vessels possibly via a novel mechanism that involves the opening of K^+ ^_ATP _channels [13]. The role of H_2_S as an inflammatory mediator is supported by the pharmacological inhibition of H_2_S biosynthesis by the CSE enzyme inhibitor D,L-propargylglycine or β-cyano-L-alanine in conditions such as acute pancreatitis, haemorrhagic shock and endotoxemia [14-16]. H_2_S as a smooth muscle relaxant has been investigated in various smooth muscle tissues. The H_2_S donor sodium hydrosulphide (NaHS) relaxed guinea pig and rat ileum smooth muscle and also the thoracic aorta and portal vein [11,17]. Sidhu *et al*. [18] showed that NaHS relaxed isolated pregnant rat uterine strips in vitro, demonstrating the role of H_2_S as a smooth muscle relaxant. However the study did not investigate the production of H_2_S or the expression of the CBS and CSE enzymes in intrauterine tissues, and this has not been reported previously.

The aim of the present study was to investigate the endogenous production of H_2_S and identify the expression of CBS and CSE enzymes in rat and human intrauterine tissues.

## Methods

### Tissue collection

Tissue was collected from 36 pregnant Sprague-Dawley rats (175–250 g) at 19 days of gestation and from 4 non-pregnant Sprague-Dawley rats in the proestrous stage of the oestrous cycle, in accordance with the Home Office Guidance on the Operation of Animals (Scientific Procedures) Act 1986. Rat tissue was collected at 19 days of gestation as samples were required close to term, but not at term, which corresponds to a time frame in human pregnancy when premature labour or pre-eclampsia could occur. In pregnant rats, the uterus was incised and samples of the amnion were dissected from the complete amniotic sac, placentas were then removed from their attachment sites and cleaned of any fetal membrane attachments. Finally, whole samples of uterus were dissected out and retained. In non-pregnant rats samples of the whole uterus were dissected out. Samples of liver were retained from each animal in order to provide positive controls. All tissue samples were washed with sterile saline to remove excess blood.

Human placentas with attached amnion and myometrial samples were collected at four elective Caesarean sections at term from normal pregnancies with informed consent from the Women's Unit at University Hospital Coventry and Warwickshire, with ethical approval from Coventry Local Research Ethics Committee. Samples of amnion were removed from the placentas and 2 cm core samples of the placenta were obtained. Small samples of myometrium were retained during the Caesarean section procedure. Unfortunately, samples from pre-eclamptic placentas were not available in sufficient numbers for statistical comparison. All tissue samples were washed with sterile saline to remove excess blood and were stored at -80°C until use.

### Endogenous production of H_2_S

The endogenous production of H_2_S was measured using the methylene blue assay method of Zhao *et al*. [12] with modifications. Ten grams of tissue were homogenised in 6–9 ml of ice-cold 50 mM potassium phosphate buffer (pH 6.8). Fifty percent tissue homogenate was added to ten 30 ml capacity universal containers loaded with a reaction mixture of 100 mM potassium phosphate buffer (pH 7.4), 2 mM pyridoxal 5'phosphate and 10 mM L-cysteine. Small plastic test tubes were used as centre wells containing 1% zinc acetate (0.5 ml) with a 5.5 cm filter paper to trap evolved H_2_S. Septum lids were placed on the universal tubes which were then transferred to a shaking water bath at 37°C and incubated for 6 hours. The reaction was stopped after 6 hours by the injection of 50% trichloroacetic acid (0.5 ml) via the septum lid into the reaction mixture. To ensure maximum trapping of evolved hydrogen sulphide the reaction was allowed to incubate for a further 1 hour in the shaking water bath. The methylene blue assay was used to measure the sulphide concentration of the centre well contents. The contents of the centre wells were transferred to test tubes and the following were added to each tube: 3.5 ml of distilled water, 0.4 ml of N,N-dimethyl-p-phenyldiamine sulphate (20 mM in 7.2 M HCl) and 0.4 ml of ferric chloride (30 mM in 1.2 HCl). The tubes were incubated at room temperature for 20 minutes. The absorbance was measured at 670 nm on a spectrophotometer (Cecil, Cambridge, UK). Calibration standards were prepared using a 10 mM stock solution of NaHS to produce a standard calibration curve. Sulphide concentrations were measured and the production rate of H_2_S was calculated. The experiment was run in parallel without L-cysteine in the reaction mixture, as a negative control. The effect of NO on H_2_S production was investigated using the NO donor sodium nitroprusside (SNP) (Sigma-Aldrich, Poole, UK). The reaction mixture was incubated with 1 μM of SNP. The production of H_2_S under low oxygen conditions was investigated by flushing nitrogen gas into the universal tubes containing reaction mixture before sealing with septum lids. The concentration of oxygen in the reaction tubes was not measured. All experimental runs involved ten replicates.

### Western blotting

The expression of CBS and CSE was investigated in rat and human intrauterine tissues using Western blotting. Tissue was lysed in lysis buffer and left to incubate on ice for 60 minutes. Supernatants were retained by centrifugation at 14,000 g for 20 minutes at 4°C. Supernatant samples (50 μg/μl) were prepared for Western blot with loading buffer. Samples were separated on a 10% SDS-PAGE gel for 55 minutes at 150 V. The transfer of proteins was checked with Ponceau stain (Sigma, Poole, UK). The SDS-PAGE gel was transferred to PVDF membrane (Amersham, Buckinghamshire, UK) at 50 V for 2 hours. 5% non-fat dried milk was used to block the membrane. The primary antibodies for CSE and CBS were home made at Lakehead University (Canada). The membrane was then incubated with the primary antibody (dilution 1:500) overnight at 4°C. Primary antibody was removed by three 5 minute washes with TBST. The membrane was incubated with secondary antibody (dilution 1:5000 for both goat anti-mouse and goat anti-rabbit (Abcam, Cambridge, UK) for 1 hour at room temperature. Secondary antibody was removed by three 5 minute washes with TBST. The ECL Plus Western Blotting detection kit (Amersham, Little Chalfont, UK) was used to detect the presence of the enzymes using the ChemiDoc EQ imager (Bio-Rad, Hemel Hempstead, UK). The PVDF membranes were reprobed with the loading control β-actin based on the method used by Liao *et al*. [19] with some modifications. After incubation with the ECL Plus Western Blotting detection kit, the PVDF membranes were kept in fresh TBST at 4°C for 24 hours. The PVDF membranes were washed three times for 5 minutes with TBST. These membranes were reprobed with β-actin antibody (Abcam, Cambridge, UK) (dilution 1:1000) in 5% milk (non-fat dried milk) overnight. The ECL Plus Western Blotting detection kit was used to detect the presence of β-actin. Rat kidney was used as a positive control for CBS, as it has previously been detected in rat kidney [20]. For CSE the positive control used was rat aorta. Non-pregnant (NP) rat uterus was also investigated for the expression of both CBS and CSE (n = 4).

The following rat tissues were investigated: uterus, placenta and fetal membranes (amnion) (n = 4). Human tissues investigated were: chorion, amnion, myometrium and placenta (n = 4). The Western blotting technique used was qualitative.

### Statistical analysis

H_2_S production rates in nM min^-1 ^g^-1 ^wet tissue are shown as mean ± SD. Mean production rates were compared using Student's T test for unrelated samples or ANOVA with a LSD posthoc test corrected for repeated tests, as appropriate. P < 0.05 was considered statistically significant.

## Results

### Assay validation

The coefficient of variation for the methylene blue assay was 5.7%. The intrapair correlation was 0.975 (P < 0.001).

### Basal production of H_2_S in rat liver, rat intrauterine tissues and human placenta

Rat intrauterine tissue and human placenta homogenates produced H_2_S from L-cysteine in vitro (Table 1). Rat liver homogenate (positive control) produced H_2_S at a much higher rate than the rat intrauterine tissues (Table 1). The order of H_2_S production rates was rat liver > human placenta > rat uterus > rat fetal membranes > rat placenta (Table 1).

**Table 1:** Comparison of mean production rates of H_2_S from homogenates of rat liver, rat intrauterine tissues and human placenta (nM/min/g) in the presence and absence (control) of 10 mM L-cysteine.

Tissue	Treatment	H_2_S production rate (nM/min/g)			Significance
		Mean	SD	n	
Rat liver	Control	4.0	3.5	4	
	L-cysteine	777	163	4	P < 0.05
					
Rat uterus	Control	0	0	4	
	L-cysteine	168	100	4	P < 0.05
					
Rat placenta	Control	0	0	4	
	L-cysteine	11.1	4.7	4	P < 0.05
					
Rat fetal membranes	Control	0	0	4	
	L-cysteine	22.3	15.0	4	P < 0.05
					
Human placenta	Control	0	0	4	
	L-cysteine	200	102	4	P < 0.05

A value of 0 indicates below the level of detection.

### Endogenous production of H_2_S in the presence of a nitric oxide donor

For rat liver there was a trend of increased production of H_2_S in the presence of the NO donor, however the difference in mean production rates did not reach significance (Table 2). A similar trend was observed for rat uterus and rat placenta homogenates (Table 2). For rat fetal membranes H_2_S production was significantly elevated in the presence of the NO donor (p < 0.05) (Table 2).

**Table 2:** Effect of a nitric oxide donor on mean production rates of H_2_S from homogenates of rat liver and intrauterine tissues (nM/min/g) in the presence and absence (negative control) of 10 mM L-cysteine.

Tissue	Treatment	H_2_S production rate (nM/min/g)			Significance
		Mean	SD	n	
Rat liver	-ve control	4.0	3.5	4	
	L-cysteine	575	172	4	
	+ NO donor	596	174	4	NS
					
Rat uterus	-ve control	0	0	4	
	L-cysteine	438	277	4	
	+ NO donor	433	224	4	NS
					
Rat placenta	-ve control	0	0	4	
	L-cysteine	45.7	51.6	4	
	+ NO donor	51.6	26.2	4	NS
					
Rat fetal membranes	-ve control	0	0	4	
	L-cysteine	43.4	20.3	4	
	+ NO donor	57.4	31.5	4	P < 0.05

A value of 0 indicates below the level of detection.

### Endogenous production of H_2_S under low oxygen conditions

Under low oxygen conditions production of H_2_S was significantly increased compared to room air oxygen conditions for rat liver, uterus and fetal membranes (P < 0.05), but not rat placenta (Table 3). For human placenta there was a significant increase in H_2_S production under low oxygen conditions (P < 0.05) (Table 3).

**Table 3:** Effect of a low oxygen environment on mean production rates of H_2_S from homogenates of rat liver, rat intrauterine tissues and human placenta (nM/min/g) in the presence and absence (negative control) of 10 mM L-cysteine.

Tissue	Treatment	H_2_S production rate (nM/min/g)			Significance
		Mean	SD	n	
Rat liver	-ve control	4.0	3.5	4	
	L-cysteine	331	269	4	
	Low O_2_	454	205	4	P < 0.05
					
Rat uterus	-ve control	0	0	4	
	L-cysteine	405	432	4	
	Low O_2_	606	620	4	P < 0.05
					
Rat placenta	-ve control	0	0	4	
	L-cysteine	20	10.9	4	
	Low O_2_	17.1	11.2	4	NS
					
Rat fetal membranes	-ve control	0	0	4	
	L-cysteine	146	120	4	
	Low O_2_	490	218	4	P < 0.05
					
Human placenta	-ve control	0	0	4	
	L-cysteine	200	102	4	
	Low O_2_	397	326	4	P < 0.05

A value of 0 indicates below the level of detection.

### Expression of CBS and CSE in rat intrauterine tissues

**Figure 1:**
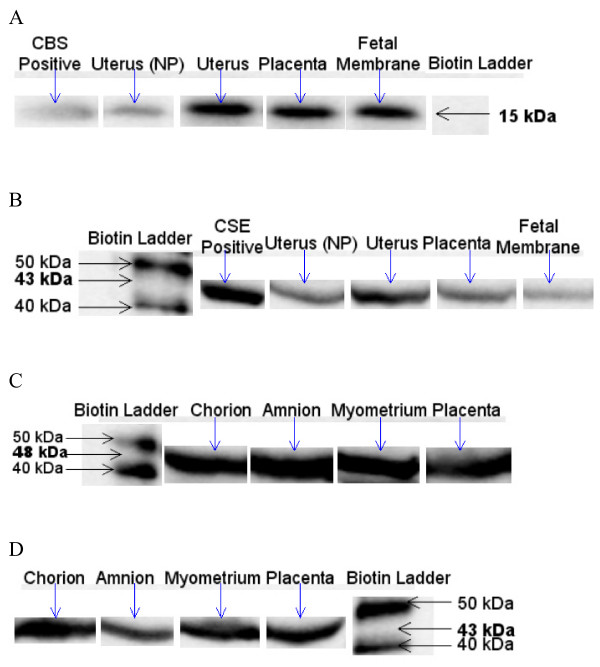
**Expression of CBS and CSE enzymes in rat and human intrauterine tissues**. A Expression of CBS at 15 kDa in rat intrauterine tissues. Expression of CBS was detected in pregnant and non-pregnant rat uterus, placenta and fetal membranes. B Expression of CSE at 43 kDa in rat intrauterine tissues. Expression of CSE was detected in pregnant and non-pregnant rat uterus, placenta and fetal membranes. C Expression of CBS at 48 kDa in human intrauterine tissues. Expression of CBS was detected in human chorion, amnion, myometrium and placenta, D Expression of CSE at 43 kDa in human intrauterine tissues. Expression of CSE was detected in human chorion, amnion, myometrium and placenta.

Figure 1 shows the expression of CBS in rat intrauterine tissues. Expression of CBS was detected at 15 kDa in rat tissues (Fig. 1a), while expression of CSE was detected at 43 kDa in all tissues investigated (Fig. 1b). β-actin was used as a loading control for each Western blot and was detected at 42 kDa.

### Expression of CBS and CSE in human intrauterine tissues

Expression of CBS was detected at about 48 kDa in all human tissue samples (Fig. 1c). The same human samples were used to investigate the expression of CSE in intrauterine tissues. CSE expression was detected at 43 kDa in all human tissues (Fig. 1d). β-actin was used as loading controls for these Western blots and expression was detected at 42 kDa.

## Discussion

### Endogenous production of H_2_S in intrauterine tissues

The physiological role of H_2_S in the reproductive system has not been fully investigated. Sidhu *et al*. [18] previously showed that L-cysteine and NaHS relaxed pregnant rat uterus in vitro, however the endogenous production of H_2_S in intrauterine tissue was not investigated in that study. In the present study we are the first to report the production of H_2_S in rat and human intrauterine tissues. The baseline production of H_2_S in rat intrauterine tissues was established in the present study. The results showed that in the absence of additional L-cysteine all rat intrauterine tissues produced very low levels of H_2_S, the greatest value was for rat liver (4.01 ± 3.5 min^-1^g wet tissue^-1^). The tissue homogenates would have contained some blood and a small concentration of endogenous L-cysteine would have been present. It is also possible that when the homogenates were acidified with trichloroacetic acid some sulphide ions present were driven off as H_2_S.

With the addition of 10 mM L-cysteine a significant increase in the production of H_2_S was observed in all tissues. Rat liver produced H_2_S at a significantly higher production rate in comparison to all intrauterine tissues; this is possibly due to the high expression of CSE in rat liver [6,7]. The present study showed that rat uterus produced H_2_S at a greater rate than rat placenta and fetal membranes; the lowest production rate was observed in rat placenta. Human placenta exhibited significant H_2_S production. The present study showed that H_2_S can be produced from L-cysteine in intrauterine tissues.

### The endogenous production of H_2_S in the presence of a nitric oxide donor

Earlier studies have demonstrated the effect of NO on the production of H_2_S. Zhao *et al*. [12] demonstrated that NO up-regulated the production of H_2_S. In the present study comparison of treatments of L-cysteine and the NO donor showed no significant difference in the production of H_2_S, showing that the NO donor did not affect the production of H_2_S. Similar results were observed with rat uterus and placenta. In rat fetal membranes the presence of the NO donor showed a significantly (P < 0.05) elevated production rate in comparison to the L-cysteine treatment alone (Table 2). A similar result was observed by Zhao *et al*. [12] where the production of H_2_S was up-regulated by SNP in rat aortic tissue. Zhao *et al*. [12] demonstrated that NO up-regulated the production of H_2_S via the cGMP pathway. The mechanism by which NO affects the H_2_S production rate was not investigated in this study. It is not clear why the augmenting effect of SNP only reached significance in rat fetal membranes.

### The endogenous production of H_2_S under low oxygen conditions

The present study is the first to demonstrate the increase in measured production of H_2_S under low oxygen conditions in rat and human intrauterine tissues. The production rate of H_2_S was significantly increased (P < 0.05) in rat liver under low oxygen conditions. Similar results were observed for rat uterus (Table 3). For rat placenta there was no significant difference in the production rates of H_2_S under normal and low oxygen conditions. Rat fetal membranes followed the same trend as rat liver and rat uterus under normal and low oxygen conditions. Under low oxygen conditions the production rate of H_2_S was significantly elevated (P < 0.05). The present study also investigated the endogenous production of H_2_S in human placenta under normal and low oxygen conditions. It is not clear why a significant increase was seen for human placenta, but not rat placenta. A significant (P < 0.05) increase in H_2_S production was observed in human placenta under low oxygen conditions. Low oxygen conditions were used as hypoxia is associated with pre-eclampsia, where poor placental function can reduce the supply of oxygen and nutrients to the fetus resulting in intrauterine growth restriction (IUGR) and other placental dysfunctions. Hypoxia can also bring about other pre-eclamptic features such as the release of proinflammatory cytokines and oxidative stress [21-23]. Hypoxic conditions can also reduce the uteroplacental perfusion, which may lead to inflammatory conditions i.e. oxidative stress [23]. In the present study we found that the endogenous production of H_2_S under low oxygen conditions was elevated in rat uterus, fetal membranes and in human placenta in vitro. It is possible that under atmospheric oxygen conditions some oxidation of evolved H_2_S could occur, thus reducing the amount of trapped H_2_S in the assay. Under low oxygen conditions perhaps less H_2_S was oxidised, leading to increased measured production rates of H_2_S. However, if it were a simple chemical effect then it would be expected to affect all H_2_S production measurements equally, but this was not the case (Table 3). For example, measured H_2_S production from rat placenta was not increased under low oxygen conditions. It seems more likely that the CBS and CSE enzymes could be directly affected by oxygen. CBS contains heme in its structure, which could bind oxygen and affect enzyme function. In comparison, CSE does not contain heme so an alternative or novel mechanism could be involved. Clearly there is a difference between low oxygen incubation for tissue homogenates in vitro and physiological hypoxia in vivo. The enzyme function of CBS and CSE appear to be affected by oxygen levels. It is possible that increased production of H_2_S under hypoxic conditions could have a role in the pathology of pre-eclampsia.

### Expression of cystathionine β-synthase (CBS) and cystathionine γ-lyase (CSE)

The human CBS gene has been mapped to chromosome 2 and contains 23 exons. Exons 1–14 and 16 encode the CBS enzyme. The molecular weight of CBS is 160,000, and in human and rat liver the primary translational product of the CBS gene gives rise to tetrameric subunits of 63 kDa. These subunits are composed of 551 amino acids residues, and the enzyme also contains the pyridoxal 5'-phophaste and haem molecule per subunit essential for its activity [24,25]. The haem molecule could be the direct target of NO as it can bind to haem with high affinity. The proteolytic cleavage of the 63 kDa subunit yields a 48 kDa dimer subunit (40–413 amino acid residue), which is accompanied by a 60-fold increase in the enzyme's specific activity with physiological concentrations of homocysteine [24-27].

The presence of CSE has previously been found in rat liver and in a variety of species including: *Neurospora crassa, Aspergillus nidulans, Saccharomyces cerevisiae and Saccharomycopsis lipoltic *[28-30]. CSE has a molecular weight of 166 kDa. Like rat CBS, rat CSE is also composed of 4 identical tetrameric subunits at 43 kDa and require pyridoxal 5'-phophaste for their activity [28,31]. The cDNA sequence of rat CSE in comparison to related *E. coli *enzymes (cystathionine γ-synthase and cystathionine β-lyase) share a common ancestral gene as well as identical tetrameric subunits at 43 kDa. These enzymes from *E. coli *are also pyridoxal 5'-phophaste dependent [6]. Expression of CSE is mainly abundant in vascular tissues, while expression is increased in fetal liver in later stages of development [9,28].

The expression of CSE and CBS has previously been reported in mammalian tissues. CBS is the main H_2_S producing enzyme in brain tissue [8], while CSE is responsible for H_2_S production in vascular tissues [9].

The presence of CBS and CSE has not been previously reported in rat or human intrauterine tissues. In the present study, the expression of CBS and CSE was detected in rat and human intrauterine tissues qualitatively by Western blotting. CBS was detected at 15 kDa in rat kidney (positive control), non-pregnant uterus, pregnant uterus, placenta and fetal membranes (Fig. 1a). It is not clear why a fragment of CBS was detected at 15 kDa in rat tissue, but at 48 kDa in human tissue. Skovby et al.(1984) [24] previously reported a 15 kDa peptide cleaved from CBS by proteolysis. As the primary antibody used was polyclonal it is possible that it bound to an epitope on the 15 kDa fragment from rat CBS. CSE was detected at approximately 43 kDa in rat aorta (positive control), non-pregnant uterus, pregnant uterus, placenta and fetal membranes (Fig. 1b). These results are in accordance with Cheng *et al*. [9] who previously reported the expression of CSE in rat vascular tissues at 43 kDa.

The present study also detected the presence of CBS and CSE in human intrauterine tissues. Expression of CBS was detected in human chorion, amnion, myometrium and placenta (Fig. 1c). A band at 48 kDa was detected in all tissues, suggesting CBS expression. However, these results do not agree with the previous findings by Ratnam *et al*. [32] who reported expression of CBS at 63 kDa in rat liver cells. It has been previously reported that rat liver CBS has a subunit at 48 kDa which can be produced by proteolytic cleavage of the 63 kDa subunit [7,24]. The present study detected a band at about 48 kDa, which is likely to be the subunit expression at 48 kDa of CBS. The present study also investigated the expression of CSE in human chorion, amnion, myometrium and placenta. The results detected expression of CSE at 43 kDa in all human tissues (Fig. 1d). These results agree with similar findings of CSE expression detected at 43 kDa in rat vascular tissues [9].

This is the first study to report the detection of both CBS and CSE in rat and human intrauterine tissues and the production of H_2_S by these tissues. The results demonstrated the endogenous production of H_2_S in rat and human intrauterine tissues via CBS and CSE enzymes.

## Conclusion

Basal production of H_2_S was demonstrated in rat uterus, placenta, fetal membranes and human placenta. The endogenous production of H_2_S was up-regulated by the NO donor SNP in rat fetal membranes. Exposure of cell homogenates from rat liver, uterus, fetal membranes and human placenta, to low oxygen levels increased H_2_S production rates. The presence of CBS and CSE enzymes was demonstrated, for the first time, in rat and human intrauterine tissues. Endogenously produced H_2_S could possibly have a role in the pathology of pre-eclampsia, however further investigation of the role of H_2_S in the reproductive system is required.

## Competing interests

The authors declare that they have no competing interests.

## Authors' contributions

PP conducted the laboratory work, analysed the data and drafted the paper. MV provided human tissue and ethical approval. JH provided biochemical advice and reviewed the draft paper. RW provided the primary antibodies, gave advice and reviewed the draft paper. RJC designed the study and amended the paper. All authors read and approved the final manuscript.
